# Optimal pooling strategies for respiratory virus testing: A comparative cost-effectiveness analysis

**DOI:** 10.1371/journal.pgph.0006646

**Published:** 2026-07-16

**Authors:** Fan Zhong, Changyu Ni, Bingshun Wang

**Affiliations:** 1 Ulink College of Shanghai, Shanghai, China; 2 Institute of Clinical Medicine, Rujin Hospital Luwan Branch, Shanghai Jiao Tong University School of Medicine, Shanghai, China; PLOS: Public Library of Science, UNITED STATES OF AMERICA

## Abstract

Pooled testing represents a cost-efficient strategy for large-scale respiratory virus screening. However, determining the optimal pool size (OPS) across varying prevalence rates and diagnostic performance metrics remains a critical challenge in respiratory virus surveillance. We evaluated four hierarchical OPS algorithms, all employing the original solution method (OSM), and proposed a modified solution method (MSM) based on objective function optimization. Through Monte Carlo simulations and logit modeling, we generated COVID-19 infection data representative of community transmission patterns. These data were analyzed using a comparative cost-effectiveness framework to assess OSM and MSM approaches. Our analysis across various prevalence rates (0.1-30.0%), sensitivities (0.8-1.0), and specificities (0.97-1.00) revealed Hanel et al.‘s algorithm consistently yielded the largest OPS values under OSM. MSM revealed minimal deviations from OSM in most scenarios, though it effectively corrected Kim et al.’s inflated OPS values at high prevalence (~30%) with low sensitivity/specificity. Three algorithms produced comparable OPS configurations, outperforming OSM. Hanel’s and Regen’s algorithms emerged as the most cost-effective options, with Hanel’s method being optimal for low additional costs in second-stage testing and Regen’s for high additional costs. MSM significantly reduced inter-algorithm cost differences compared to OSM. This study provides a comprehensive evaluation of OPS determination algorithms in pooled PCR testing for respiratory viruses, demonstrating robust OPS configurations and enhanced cost-effectiveness through MSM implementation. The proposed MSM addresses existing limitations in pooled testing strategies, facilitates efficient resource allocation, and contributes to improved respiratory virus surveillance and pandemic response.

## Introduction

The Coronavirus Disease (COVID-19) pandemic has emerged as a global health crisis, with the World Health Organization (WHO) reporting a cumulative total of over 777 million confirmed infections and 7 million fatalities worldwide by 19 January 2025 [1]. Various aspects of social life, economic status, and healthcare systems were significantly affected by the pandemic [2]. Many infectious diseases, not limited to COVID-19, have seasonality [3], indicating long-term potential challenges and threats to humanity.

Infectious diseases spread through physical contact networks and continuously affect all human life. Therefore, implementation of early prevention and control measures for disease outbreaks is important [4]. Nowadays, the reverse problem of propagation, named source identification, is gaining increasing popularity in order to control their spread effectively [5]. Current diagnostic approaches for COVID-19 encompass etiological, serological, and radiological methodologies, among which quantitative polymerase chain reaction (PCR) and DNA sequencing are the gold standards for pathogen detection [6].

During the COVID-19 pandemic, pooled PCR testing has been a widely adopted strategy for infection screening and is one of the most efficient aapproaches [7]. This testing protocol, originally proposed by Dorfman in 1943 [8], involves the aggregation of specimens from multiple individuals into a single testing pool for simultaneous laboratory analysis. The pool size, defined as the number of individual samples combined in a single test, determined the total number of tests required and the associated diagnostic costs. Consequently, an optimal pool size (OPS) is key for pooled PCR testing to maximize cost-effectiveness [8].

Through a comprehensive literature review, we identified four distinct mathematical models for determining the OPS using hierarchical pooled testing protocols [9–12]. However, a comparative analysis of these models under empirical prevalence rates with defined testing sensitivities and specificities remains unexplored in existing research. We conducted a cost-effectiveness analysis of community-based two-stage pooled PCR testing to analyze the potential differences among these models and provide evidence-based recommendations for healthcare policymakers. In a community-based context, family members are inherently more likely to disseminate the disease to each other; therefore, pooling homogeneous individuals is a logical approach to reduce the cost of testing [7,13,14]. In our study, we implemented a family grouping strategy, wherein specimens from household members were preferentially pooled during sample collection. Owing to the unavailability of real-world COVID-19 infection data, the analysis was conducted using simulated data generated using a computer program.

## Methods

### Model design

#### Monte carlo simulation.

Monte Carlo simulations are often used to generate random samples from probability distributions. In this study, we used it to model family size distribution, which was considered to follow a zero-truncated Poisson distribution, a discrete probability distribution conditional on a Poisson-distributed random variable given that the value of the random variable excludes zero [15], with mean $\mu =\frac{\lambda }{\left(\text{1}-e^{-\lambda }\right)}$ and variance $\sigma^{\text{2}}=\frac{\lambda +\lambda^{\text{2}}}{\left(\text{1}-e^{-\lambda }\right)}-\frac{\lambda^{\text{2}}}{{\left(\text{1}-e^{-\lambda }\right)}^{\text{2}}}$.

#### Logit model.

The logit model establishes a mathematical relationship between the log odds of an event of interest and a linear predictor composed of one or more independent variables, providing a quantitative approach to assess the transmission risk and infection probabilities among household contacts following the primary case [16]. Thus, the primary case infection can be simulated as:


$$\begin{array}{c} ogit\left(P(Infection=\text{1})\right)=\beta_{\text{0}}, \end{array}$$

where $\beta_{\text{0}}$, the logit intercept, denotes the expected prevalence among the population. Secondary infections in primary cases can be described as follows:


$$\begin{array}{c} ogit\left(P\left({Infection}^{\left(i\right)}=\text{1}\right)\right)=\beta_{\text{0}}+\beta_{\text{1}}{X_{\text{1}}}^{\left(i\right)}, \end{array}$$

where $\beta_{\text{1}}$ denotes the effect of primary case infection status on the secondary infection outcome of other members within a family. The estimation of $\beta_{\text{1}}$ was largely obtained by searching for relevant published articles.

With simulated data available on the independent variable $X_{\text{1}}$ and an estimate of $\beta_{\text{1}}$, an iterative bisection procedure [17] was used to ascertain the requisite value of the parameter $\beta_{\text{0}}$. This was repeated until the gap between the empirical prevalence based on the simulated data and the expected prevalence was less than the prespecified tolerance level, which was set at 1% of the corresponding expected prevalence (see S1 Table).

#### Algorithms to obtain optimal pool sizes.

Our pooled testing protocol was based on the Dorfman procedure [8], which is the foundational methodology for group testing strategies [7]. Two-stage testing involved equal-sized pools in the first stage, followed by individual diagnostic testing of all samples if the pool tested positive. Therefore, determining an OPS is critical to achieving cost-efficiency and minimizing testing expenditures [8,18].

We compared four different algorithms to identify OPSs. Although all algorithms share the common objective of minimizing the expected number of tests per individual, denoted as E(T), at the OPS, they use different mathematical approaches and computational frameworks. Table 1 summarizes the simplified objective functions of each algorithm. The mathematical models, formulas, derivations, and explanations are provided in S1 File.

**Table 1:** Summary for objective functions of four algorithms.

Algorithms	Objective functions E(T)
D.A. Caqueo et al. [9]	$f(n)=\frac{\text{1}}{n}+\text{1}-(\text{1}-p{)}^{n}-S$
F. Regen et al. [10]	$g(n)=\frac{\text{1}}{n}+\text{1}-(\text{1}-p{)}^{n}-S$
H.Y. Kim et al. [11]	$s(n)=\frac{\text{1}}{n}+\left(\text{1}-S_{e}-S_{p}\right)(\text{1}-p{)}^{n}+S_{e}$
R. Hanel et al. [12]	$t(n)=s(n)+\left(S_{e}+S_{p}-\text{1}\right)S$

Notes: S=∑k=1n(kn)pk(1−p)n−k(1−Se)kSpn−k; p denotes the disease prevalence; $S_{e}$ and $S_{p}$ denotes the testing sensitivity and specificity respectively; n denotes the pool size.

#### A modified approach to solving OPS.

Among the algorithms mentioned above, Caqueo et al. and Kim et al. calculated objective function values repetitively for many possible pool sizes and reported the OPSs that minimized the objective function value, whereas Regen et al. and Hanel et al. determined the OPSs by solving for the derivatives of objective functions equal to zero, which correspond to the minima of the objective functions. Although the differentiation method promotes computational efficiency, we still consider it inappropriate to round the numerical solutions to the nearest integer because OPS can only take integer values in practice. We developed a hybrid optimization strategy to address this limitation that integrates both methodologies. Initially, we obtained the analytical solution through differentiation, followed by a comparative evaluation of the two adjacent integers. The integer value that yielded the minimal objective function value was subsequently selected as the final OPS.

#### Cost-effectiveness analysis.

Cost-effectiveness analysis is an economic analysis that quantifies costs in monetary units and outcomes in natural units. In this study, we used relative cost coefficients $\alpha_{k}$ to simplify our model and enhance its intelligibility. Given that additional time costs and transportation fees may be incurred during second-stage individual testing, the relative cost coefficient of second-stage testing $\alpha_{\text{2}}$ was computed as the ratio between mean cost per individual of second-stage to first-stage testing (see S2 File) under the assumption that $\alpha_{\text{1}}=\text{1}$ for first-stage testing. Therefore, the relative cost per individual C is expressed as


$$\begin{array}{c} C=\frac{\text{1}}{N}\sum_{k=\text{1}}^{\text{2}}{\alpha_{k}T}_{k}, \end{array}$$

where k denotes the stages of testing (k=1, 2) and N denotes the number of residents within a given community. The mean of the relative costs per individual was calculated for subsequent comparisons between the algorithms, along with its 95% confidence interval (CI), as appropriate.

Notably, we calculated $\alpha_{\text{2}}$ using the method described in S2 File during early stage of our study, where we noticed that it led to some unrealistic cost estimation under certain parameter combinations (e.g., an $\alpha_{\text{2}}$ as high as 11.94 at prevalence of 0.001 and pool size of 37). Therefore, to ensure the practicability and policy relevance of our study results, we came up with an alternative to determine the range of $\alpha_{\text{2}}$.

Specifically, we computed the threshold value for $\alpha_{\text{2}}$, above which the two-stage pooled testing protocol no longer exhibits cost-effectiveness compared to non-pooled individual testing. Thus, this threshold denotes the highest cost ratio affordable for pooled PCR testing protocols, and we would be able to select $\alpha_{\text{2}}$ values of practical implications, for subsequent cost-effectiveness analysis. In addition, we compute the area under curve (AUC) values through trapezoidal rule for $\alpha_{\text{2}}$ threshold values against prevalence for all four algorithms, to comprehensively compare their cost-effectiveness across all prevalence levels.

### Key assumptions

**Assumption 1:** The infection status(es) of other family member(s) is affected by the primary case infection within that family, given that the family size is larger than one. More risk factors could be included in logit modeling, where necessary.

**Fig 1:**
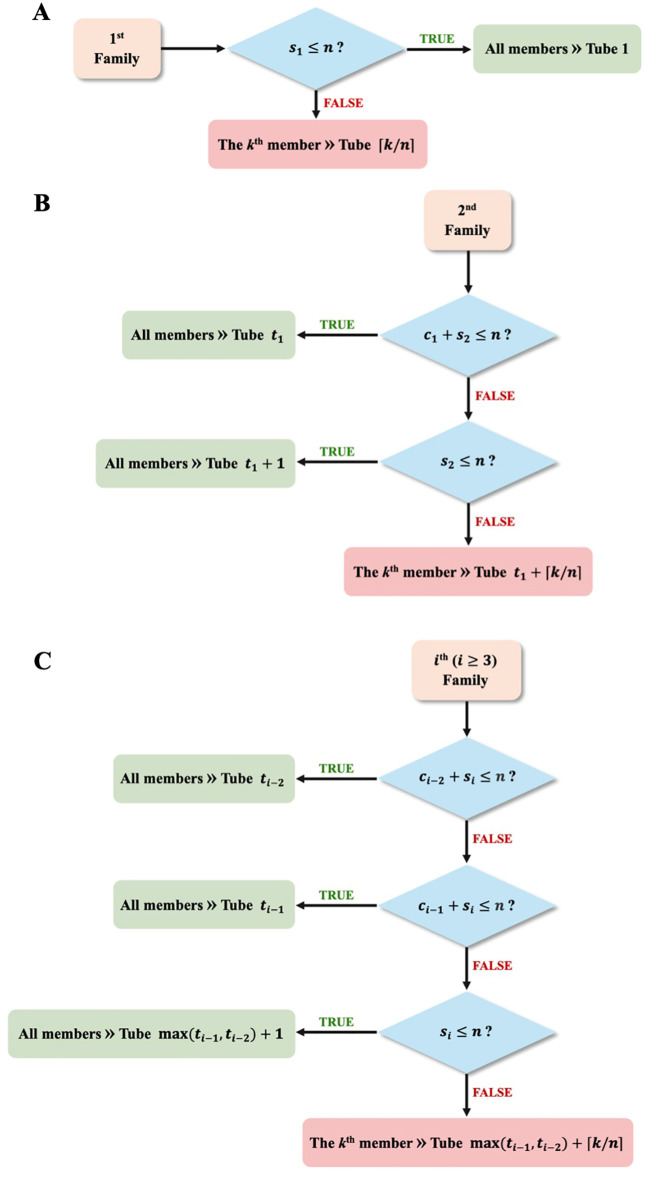
Rules for tube assignment process of (A) the 1st family, (B) the 2nd family and (C) the i^th^ family, i ≥ 3. Notes: n denotes the tube pooling size; s_i_ denotes the family size of the i^th^ family; t_i_ denotes the number of tubes used after the i^th^ family is pooled; c_i_ denotes the number of individuals in the last tube of the i^th^ family; the “⌈ ⌉” sign denotes the ceiling function, e.g., ⌈1.5⌉ = 2, ⌈3⌉ = 3.

**Assumption 2:** All member(s) from the same family are pooled into one PCR test pool unless the family size is larger than the pool size. In other words, members from the same family are separated into different tubes only if the family size exceeds the permitted pool size. The detailed rules of the tube assignment process are shown in Fig 1.

### Model input

In the Monte Carlo simulation, 5,000 independent simulation trials were conducted, with each trial comprising 2,000 distinct family units. The Poisson distribution parameter λ was specified as 3.94, based on the global average household size reported by the United Nations [19]. A case–control study reported an increased risk of secondary infection (461%) following a primary case within a family unit [20].

To conduct the cost-effectiveness analysis, we chose multiple disease prevalence rates ranging from a minimum rate of 0.1% to a maximum rate of 30.0%. For a prevalence of over 30.0%, pooled testing demonstrated no greater advantage in reducing the expected number of tests per individual [21]; thus, we set 30% as the highest feasible prevalence. The sensitivity and specificity of PCR testing ranged from 80% to 100% and 97% to 100%, respectively, according to the acceptable levels of COVID-19 diagnostic products set by the WHO [7]. A comprehensive list of these parameter values is provided in S3 Table.

### Model output

The OPSs, according to the four selected algorithms, were determined by minimizing the expected number of tests per individual for each set of disease prevalence, test sensitivity, and test specificity. Subsequently, the relative mean costs of these algorithms and their corresponding OPSs were calculated based on simulated infection data.

### Statistical analysis

The statistical analysis in this study was carried out using statistical software R 4.4.2 (*www.r-project.org*) along with the ‘tidyverse,’ ‘simstudy’ and ‘Rlab’ packages. Bootstrap percentile method [22] was used to calculate the 95% CI for the mean relative cost, implemented by the package ‘boot.’ An OPS calculator built into the R Shiny web application is available at *https://aflyingfrank.shinyapps.io/OPScalculator/*. Key codes regarding data simulation, tube ID assignment and relative cost calculation are available at *https://github.com/Aflyingfrank/Cost-effectiveness-Analysis-of-Pooled-Testing*.

## Results

### OPSs based on multiple algorithms

#### Effect of prevalence on OPS.

**Fig 2:**
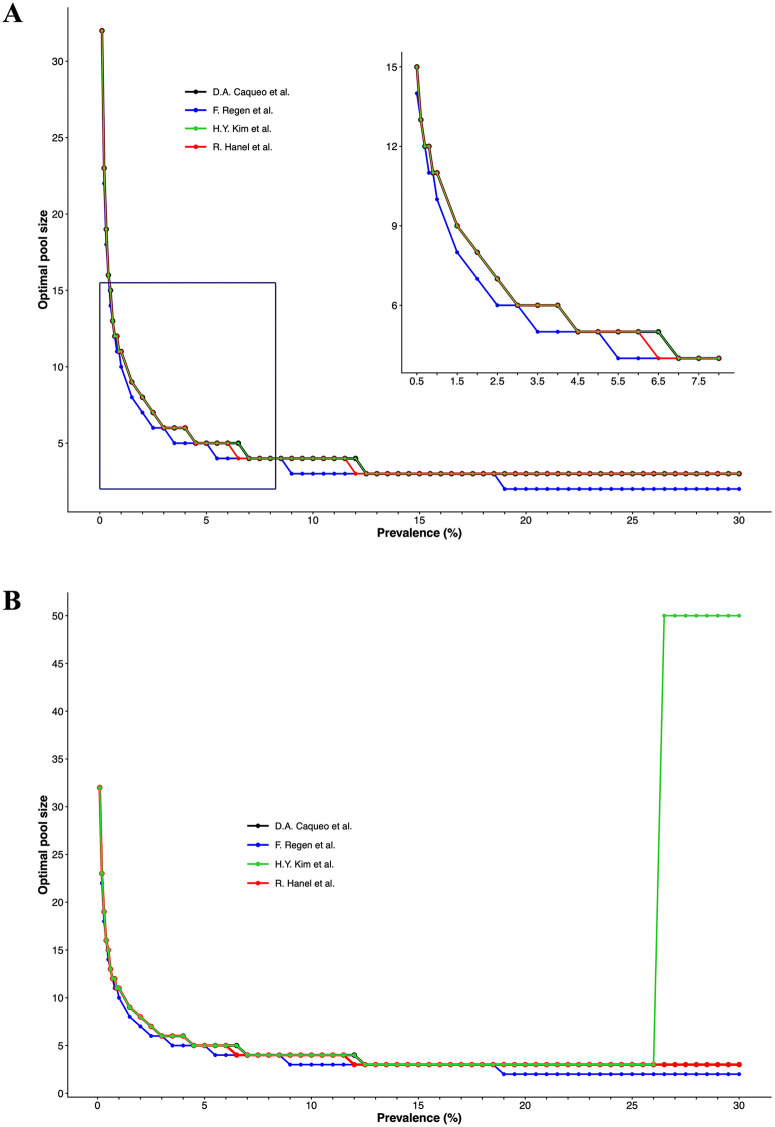
Optimal pool sizes against different prevalences at (A) sensitivity = 1 and specificity = 1, (B) sensitivity = 0.8 and specificity = 0.97.

For various sensitivity and specificity values, smaller OPSs were obtained at a higher prevalence for all four algorithms (Fig 2).

With perfect sensitivity and specificity (both equal to 1), all four algorithms derived similar OPSs over various prevalence values. Algorithms from Caqueo et al. and Kim et al. were identical throughout the prevalence trend, whereas Hanel et al. resulted in minor differences (with one unit less) at two specific prevalences (6.5% and 12.0%). Regen et al. reported the smallest OPSs (Fig 2A).

As the sensitivity and specificity decreased to the lowest acceptable level, the OPSs obtained by the algorithms of Caqueo et al. and Regen et al. were more similar and slightly lower than those of Kim et al. and Hanel et al. The latter two methods showed highly overlapping OPSs, especially with low sensitivity and specificity (Fig 2B). However, Kim et al. reported several abnormally large OPSs at a prevalence approaching 30%.

#### Effects of sensitivity and specificity on OPS.

The study showed that the three algorithms had different OPSs at different sensitivity and specificity levels, except for the algorithm proposed by Caqueo et al., whose OPSs did not differ over various sensitivity and specificity values. Furthermore, as the prevalence increased, the three algorithms’ OPSs became less sensitive to the testing sensitivity and specificity values (S1 Fig).

The OPSs from the Kim et al. and Hanel et al. algorithms were largely the same, but abnormally larger OPSs were obtained from the former when the prevalence exceeded 26% (S2 Fig). Both of these algorithms obtained larger OPSs as sensitivity and specificity decreased, while Regen et al.’s OPS increased at lower sensitivity but higher specificity and always appeared smaller than that of Hanel et al. The surface plots in S1 Fig demonstrated that sensitivity had a much greater effect on OPS than specificity across three algorithms apart from D.A. Caqueo et al.’s.

### OPSs based on OSMs and our MSM

As mentioned above, we incorporated and modified the OSMs for the OPS, given the same objective functions. When the sensitivity and specificity were equal to 1, all four algorithms gave identical OPSs when MSM was applied to their respective objective functions. Three (D.A. Caqueo et al., H.Y. Kim et al., and R. Hanel et al.) of the four algorithms obtained the same results using their OSMs. However, Regen et al.’s algorithm yielded an equal or one larger OPS with MSM than with OSM (S2 Table).

When sensitivity and specificity reached a minimum of 0.8 and 0.97, respectively, there were a similar number of differences (9 vs. 8 out of 44 OPSs) in OPSs obtained using OSM or MSM. None of the four algorithms had identical OPSs when using OSM and MSM, but almost all the differences were no more than one (larger with MSM). MSM corrected the abnormally large OPS obtained from Kim et al.’s algorithm at a prevalence of approximately 30% (Table 2).

**Table 2:** OPSs obtained from four algorithms using OSM or MSM at various prevalences when sensitivity = 0.8 and specificity = 0.97.

Prevalences (%)	D.A. Caqueo et al.		F. Regen et al.		H.Y. Kim et al.		R. Hanel et al.	
	OSM	MSM	OSM	MSM	OSM	MSM	OSM	MSM
0.1	32	32	32	32	37	37	37	37
0.2	23	23	22	23	26	26	26	26
0.5	15	15	15	15	17	17	17	17
0.8	12	12	12	12	13	14	13	13
1.0	11	11	11	11	12	13	12	12
2.0	8	8	8	8	9	9	9	9
5.0	5	5	5	5	6	6	6	6
8.0	4	4	4	4	5	5	5	5
10.0	4	4	3	4	4	5	4	4
20.0	2	3	3	3	4	4	4	4
30.0	2	3	2	3	50	3	4	4

With MSM, three of the four algorithms yielded similar OPS trends over various sensitivity and specificity levels, whereas the OPS results from the remaining algorithms showed the same trends as the OSM. Hanel et al.’s algorithm obtained larger OPSs as sensitivity and specificity decreased, while the OPSs from the other three algorithms increased at lower sensitivity but higher specificity (S3 Fig).

### Thresholds for relative cost coefficient of second-stage testing

For all four algorithms, regardless of OSM or MSM applied, the trend of $\alpha_{\text{2}}$ threshold value against prevalence behaved similar to an inversely proportional relationship, where a sharp fall was observed for prevalences below 5% and a smooth drop followed (S4 Fig and S5 Fig). For both the best and the worst sensitivity and specificity, our MSM resulted in similar AUC values. Notably, our MSM significantly raised the AUC for H.Y. Kim et al.’s algorithm at sensitivity of 0.8 and specificity of 0.97 by fixing the abnormal OPS values at high prevalence (S5 Fig).

Based on these results, we determined the range of the universal $\alpha_{\text{2}}$ values used in subsequent cost-effectiveness analysis to be from 1 to 8, in which we selected 6 distinct values (1, 1.25, 1.5, 2, 4, and 8) to investigate the trends of relative cost.

### Relative costs based on multiple algorithms

**Fig 3:**
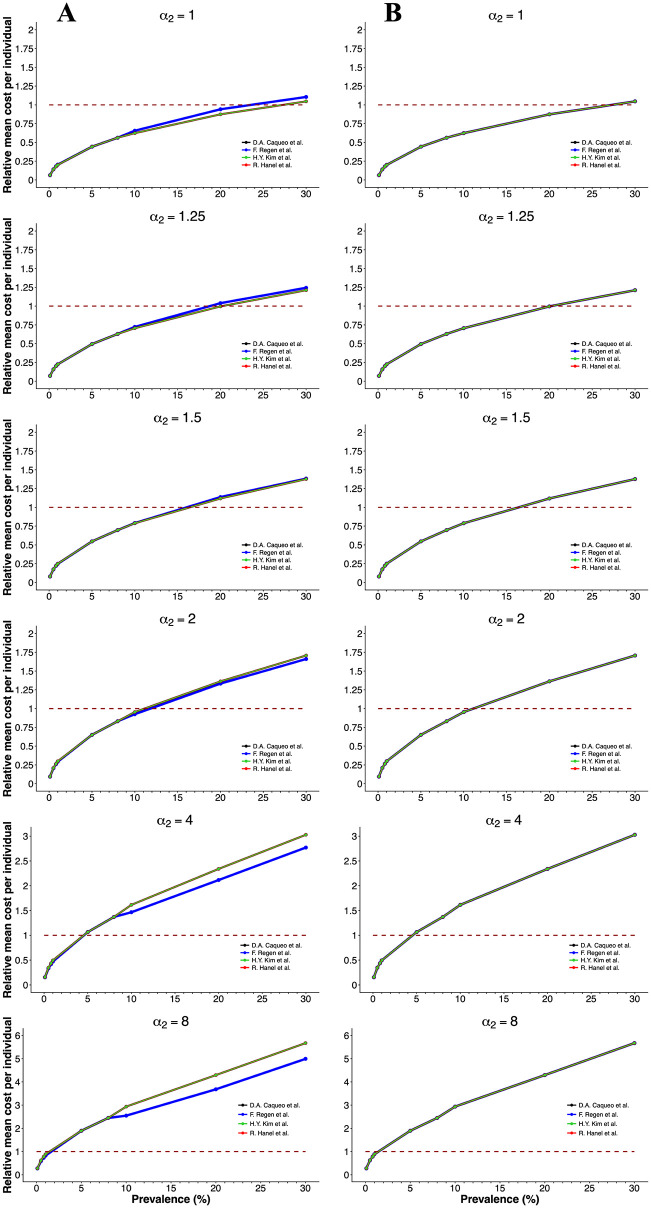
Relative costs per individual at optimal pool sizes against different prevalences at sensitivity = 1 and specificity = 1 with use of (A) original solving methods (B) our modified solving method.

**Fig 4:**
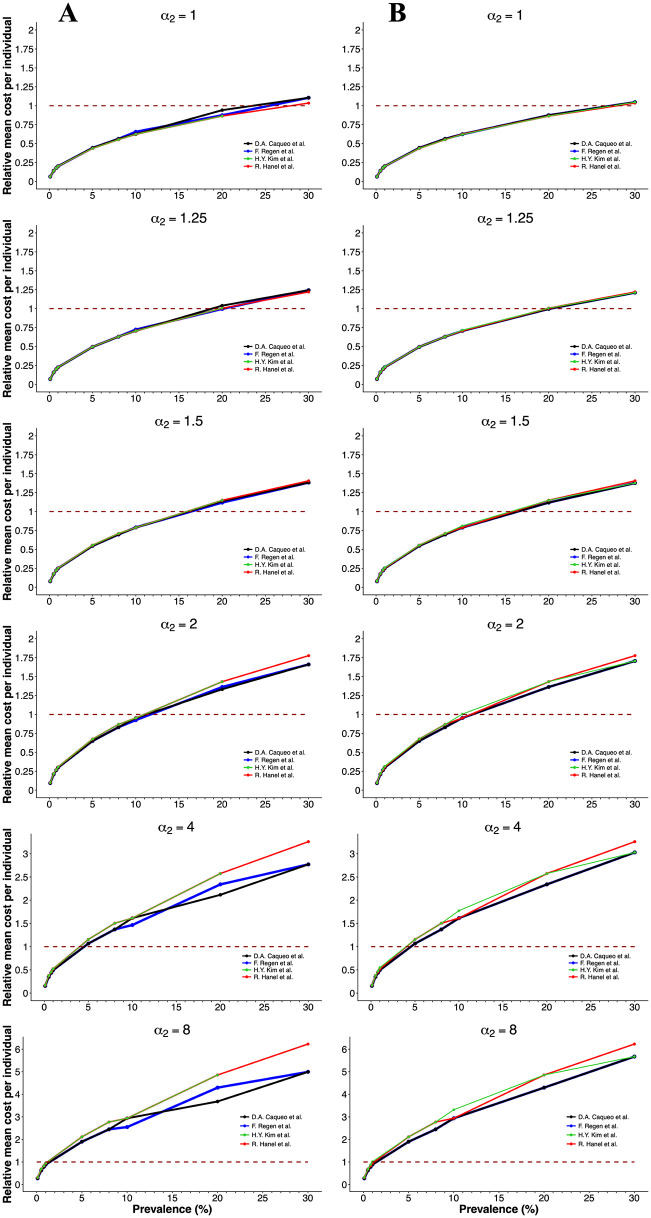
Relative costs per individual at optimal pool sizes against different prevalences at sensitivity = 0.8 and specificity = 0.97 with use of (A) original solving methods (B) our modified solving method.

The relative mean costs showed increasing trends as prevalence increased for various sensitivity and specificity values, regardless of the application of OSM or MSM (Fig 3 and Fig 4).

When both sensitivity and specificity were equal to 1, with OSM used, D.A. Caqueo et al., H.Y. Kim et al., and R. Hanel et al. obtained the lowest relative mean costs per individual (e.g., 0.0630, 95% CI, 0.0627 to 0.0633 at a prevalence of 0.1%) over all selected prevalences when $\alpha_{\text{2}}$ was equal to 1, 1.25, and 1.5, while F. Regen et al. was the most cost-effective as $\alpha_{\text{2}}$ reached 2 (Fig 3A). In contrast, all four algorithms had identical relative mean costs to the applied MSM (Fig 3B).

At the lowest sensitivity (0.8) and specificity (0.97) selected, R. Hanel et al.’s and H.Y. Kim et al.’s algorithms yielded the same minimum relative mean costs with both solution methods when $\alpha_{\text{2}}$ was 1. However, their cost advantages were reversed as $\alpha_{\text{2}}$ rose to 1.25 and above (Fig 4). The differences in the relative mean costs among the four algorithms were smaller when our MSM was used instead of the OSM.

For large values of $\alpha_{\text{2}}$ (4 and 8), the trends of relative costs and differences among algorithms amplified. In most of the cases shown above, minor or even negligible differences were noted in the relative mean cost were when the prevalence was at 8% or below.

Cost reductions achieved by our MSM compared to OSM were shown in S6 Fig. Our MSM exhibited improved cost-effectiveness when $\alpha_{\text{2}}$ is small, especially at high prevalence levels (e.g., largest cost reduction of 1.3% with prevalence of 0.8% and $\alpha_{\text{2}}$ of 1). As $\alpha_{\text{2}}$ grew as large as 2, our MSM was no longer the most cost-effective across all four algorithms.

## Discussion

To the best of our knowledge, this is the first study to directly compare four algorithms for calculating the OPS of a pooling strategy. For comparative consistency, we included the sensitivity and specificity of each mathematical algorithm, as appropriate. Using OSM, Hanel et al.’s algorithm generated the largest OPSs over the selected prevalence among all four we identified and analyzed. However, all four algorithms yielded similar OPS results when applying the proposed MSM. At their respective OPSs, Hanel et al.’s algorithm achieved the lowest relative mean costs, given that second-stage family pooling tests had a minor cost increase compared with first-stage ones. In contrast, F. Regen seemed the most cost-effective if the second-stage tests cost much more. Our proposed MSM appeared consistently optimal regarding the relative cost when applied to the objective functions of all four original algorithms. Consistent with our findings, Augenblick et al. [23] mentioned that the pooled testing of individuals with correlated risk was particularly efficient in the context of pandemics.

In theory, all four algorithms that we identified constructed their objective functions as the expected number of tests per individual; however, each used a different strategy to compute them (see S1 File for details). Caqueo et al. [9] derived it by subtracting the expected number of tests saved per individual from standard one-by-one testing, whereas Regen et al. [10] started from the total testing counts of the entire population and ended up taking its mean value. Kim et al. [11] constructed a multistage pooled testing formula by accumulating the number of tests stage by stage, which was simplified to the commonly used two-stage case. Hanel et al. [12] focused mainly on the probabilities of pool registers being positive or negative, given that each pool was replicated several times, which was tested once in this case. The objective functions of Caqueo et al. and Regen et al. were identical after simplification, and the remaining two appeared to be similar (Table 1).

Our study data revealed that at various prevalence rates, sensitivities, and specificities, the OPSs obtained from the algorithms of Kim et al. and Hanel et al. were largely matched, especially at low sensitivity and specificity. This was consistent with the behavior of the additional term $\left(S_{e}+S_{p}-\text{1}\right)S$ in Hanel et al.’s algorithm, as its value further approached 0 as $S_{e}+S_{p}$ fell. Although Caqueo et al. and Regen et al. shared the same objective function, their OSMs for the OPS varied (by iteration vs. differentiation and rounding). As a result, their OPSs showed a maximum difference of 1 unit over the range of the given prevalence, which could be interpreted as a rounding error. In contrast, the OPS trends against various sensitivities and specificities were completely different and seemingly counterintuitive. The OPS was much more sensitive to differences in sensitivities than specificities, owing to the much larger coefficient for sensitivity than specificity in the objective functions. For example, the coefficients in Kim’s algorithm were ${\text{1}-(\text{1}-p)}^{n}$ for sensitivity and $(\text{1}-p{)}^{n}$ for specificity. Nevertheless, when our MSM was applied instead of OSMs, the obtained OPSs were highly identical.

Considering the OPSs obtained over different prevalence rates, testing sensitivities, and specificities, Hanel et al. obtained the largest values among all four algorithms. The algorithm was the only one that showed almost consistent OPS results when using the OSM and our MSM. However, Kim et al.’s algorithm yielded an abnormally large OPS (e.g., a pool size of 50) with low sensitivity and low specificity when the prevalence approached 30%. The major reason for this abnormality was the application of an inappropriate OSM for its objective function. A detailed mathematical explanation is provided in S3 File.

Because Kim et al.’s algorithm calculates an objective function value for each pool size within a specified search range, it may not fit its objective function, which has oscillatory behavior with tiny variations. As a result, abnormally large OPSs were generated. Our proposed MSM computed the OPS using a combination of differentiation and discrete value matching, which better coped with the objective function and fixed the abnormality issue. All four algorithms obtained similar OPSs using our MSM, further demonstrating its applicability and robustness.

Cost reduction is a key factor in evaluating the efficiency of pooled testing [7], especially when facing health resource constraints. In our study, Hanel et al.’s algorithm obtained the largest OPSs in all cases, which could minimize the number of first-stage tests required and potentially lead to the lowest costs. However, larger pool sizes would intuitively raise the probability of a pool registering as positive, and therefore, the number of second-stage tests needed [10]. This effect of increased second-stage tests on the overall relative mean costs was rather obvious when second-stage tests cost much more than first-stage tests. As a result, Regen’s algorithm overtook Hanel et al.’s as the costs of second-stage tests increased compared to first-stage tests, becoming the most cost-effective OPS algorithm in this case. Nonetheless, with our MSM applied, Hanel et al.’s algorithm was able to significantly reduce costs under such conditions. Hanel et al.’s algorithm achieved the lowest costs at ideal sensitivity and specificity, given that second-stage testing costs were less than two times the first-stage costs. At the lowest sensitivity and specificity selected, its cost-effectiveness only lasts when the second-stage testing costs less than a quarter of the first-stage testing costs. Our MSM led to closer relative mean costs among the four algorithms by reducing the costs of less cost-effective algorithms (e.g., D.A. Caqueo et al.’s at low sensitivity and specificity). As the second-stage tests became increasingly more expensive compared to first-stage ones, our MSM’s cost-effectiveness was reduced, which implied the importance to incorporate such cost weightings ($\alpha_{\text{2}}$) into the objective functions of OPS algorithms.

Admittedly, our study has certain limitations. First, for a more concise model [24], we did not include multiple sources or cross-infections within a family (i.e., multiple individuals with first-case infections simultaneously or subsequent infections after secondary infections). Second, Hanel et al. proposed the idea of replicating a pool several times, which increases both the OPS and the expected number of tests per individual [12]. However, we did not consider this repetition during the cost-effectiveness analysis for a consistent comparison between the four identified algorithms. Third, our study only considered disease prevalence, testing sensitivity, and specificity [11] as key factors influencing OPS, while there may be more, for example, infrastructure constraints [4], the dilution effect when multiple samples are pooled [5], availability of reagents [10] and time limits before detection. Only typical values for the relative cost coefficient of second-stage testing $\alpha_{\text{2}}$ were selected in our study, while additional values could be specified accordingly in practice. In addition, we were unable to validate our findings with real-world PCR testing data, especially those with individual testing results and family IDs under various empirical infection prevalences. Finally, all four algorithms focused on two-stage pooled PCR testing in this study, which was simple, timesaving in turnarounds, [25] and widely used in a community context, although their algorithms could be extended to multiple-staged testing protocols. In contrast, the pooling testing strategy, like other adaptive and non-adaptive pooling methods, can be framed by testing centers to enhance their diagnostic capacity [7,26]. Furthermore, our findings should be applicable to other infectious diseases [27] or any other type of series testing [28].

## Conclusions

This study provides a comparative analysis of algorithms for determining the OPS in two-stage pooled PCR testing for pandemic diseases, such as COVID-19, highlighting key considerations under varying conditions. The findings demonstrate that certain algorithms consistently yield robust OPS configurations, whereas our proposed MSM enhances cost-effectiveness and optimality compared to traditional approaches. MSM addresses the limitations of existing methods in specific scenarios and offers a reliable framework for efficient resource allocation. These insights underscore the importance of selecting appropriate algorithms and solution methods to maximize the efficiency of pooled testing strategies supported by practical tools to aid decision-making and improve pandemic response efforts.

## Supporting information

S1 File Derivation of formulae for four optimal pool size algorithms. (PDF)

S2 File Description of calculation for α2 values. (PDF)

S3 File Analysis of abnormal behaviour of H.Y.Kim et al.’s algorithm. (PDF)

S1 Fig Optimal pool sizes of four algorithms against different sensitivities and specificities according to prevalence of (A) 0.1%, (B) 1%, (C) 10%. (RAR)

S2 Fig Optimal pool sizes based on H.Y. Kim et al.’s algorithm against different sensitivities and specificities at prevalence of 27%. (TIF)

S3 Fig Optimal pool sizes of four algorithms with modified solving method applied against different sensitivities and specificities according to prevalence of (A) 0.1%, (B) 1%, (C) 10%. (RAR)

S4 FigThreshold $\alpha_{2}$ values against prevalence at sensitivity = 1 and specificity = 1.(TIF)

S5 FigThreshold $\alpha_{2}$ values against prevalence at sensitivity = 0.8 and specificity = 0.97.(TIF)

S6 FigCost reduction of MSM compared to OSM against prevalence at (A)$\alpha_{2}$=1, (B)$\alpha_{2}$=1.25, (C)$\alpha_{2}$=1.5, (D)$\alpha_{2}$=2.(RAR)

S1 Table Bisection procedure to determine intercept of a logit model for outcomes with various expected prevalences, all with initial intervals of (-10, 10). (DOCX)

S2 Table OPSs obtained from four algorithms using OSM or MSM at various prevalences when sensitivity = 1 and specificity = 1. (DOCX)

S3 Table Parameters, input values and sources for cost-effectiveness analysis. (DOCX)
