# Supplementary material for: Optimal pooling strategies for respiratory virus testing: A comparative cost-effectiveness analysis
Source: PLOS Glob Public Health. 2026 Jul 16;6(7):e0006646. doi: 10.1371/journal.pgph.0006646 (PMC13375041; doi:10.1371/journal.pgph.0006646)
Supplement: S1 File — (PDF) [file pgph.0006646.s001.pdf]

Supplementary File 1

1 Preliminaries

All formulae in this supplementary file are based on two-staged hierarchical Polymerase Chain Reaction (PCR) testings. Common notations used are listed in the table 1 below.

Table 1: Common notations and their meanings

**Notations**	**Descriptions**
p	Disease prevalence, $p \in (0, 1)$
n	Pool size, $n \in \mathbb{Z}^+$
n^*	Optimal pool size, $n^* \in \mathbb{Z}^+$
S_e	Sensitivity of PCR testing, $S_e \in (0, 1]$
S_p	Specificity of PCR testing, $S_p \in (0, 1]$
$\mathbb{E}(T)$	Expected number of tests required per individual

We define $P\,(\text{negative})$ as the probability of a pool of size n registers as negative, and $P\,(\text{positive})$ as the probability of a pool registers as positive. Then $P\,(\text{negative})$ can be given by:

$$P\,(\text{negative}) = (1-p)^n + \sum_{k=1}^{n} \binom{k}{n} p^k\,(1-p)^{n-k}\,(1-S_e)^k\,S_p^{n-k}, \tag{1.1}$$

since $(1-p)^n$ gives the probability of all individuals uninfected, while the latter sum gives the probability of all infected individuals falsely registers as negative and all uninfected correctly registers as negative. Therefore, $P\,(\text{positive})$ is given by:

$$P\,(\text{positive}) = 1 - P\,(\text{negative})\,. \tag{1.2}$$

The following parts demonstrate four algorithms to find the optimal pool sizes n^*. All four pathways end up with minimizing $\mathbb{E}(T)$ when optimal pool sizes are taken. Noticeably, D.A. Caqueo et al. (2020) [1] and H.Y. Kim et al. (2007) [2] calculate $\mathbb{E}(T)$ repetitively for a number of possible pool sizes and obtain n^* which minimizes $\mathbb{E}(T)$, while F. Regen et al. (2020) [3] and R. Hanel et al. (2020) [4] solve for derivatives of objective functions equal to zero.

2 Algorithm based on D.A. Caqueo et al. (2020) [1]

Based on the number of tests saved $N_{test\ saved}$ due to pooled testing, the expected number of tests required per individual is given by:

$$\mathbb{E}(T) = 1 - N_{test\ saved} = 1 - \frac{P\,(\text{negative})\,(n-1) - P\,(\text{positive})}{n}, \tag{2.1}$$

since a negative pool will result in a test saving of $(n-1)$ while a positive pool will lead to an additional test (saving -1 tests). Hence, the optimal pool size is given as the pool size which minimizes $\mathbb{E}(T)$:

$$n^* = \arg\min_{n>1} \mathbb{E}(T). \tag{2.2}$$

3 Algorithm based on F. Regen et al. (2020) [3]

From the perspective of the entire testing population P, the expected number of tests required per individual is given by:

$$\begin{aligned}\mathbb{E}(T) &= \frac{N_{test}}{P} \\ &= \frac{N_{pool} + P\,(\text{positive}) \times N_{pool} \times n}{P} \\ &= \frac{\frac{P}{n} + P\,(\text{positive}) \times N_{pool} \times n}{P},\end{aligned} \tag{3.1}$$

where N_{test} denotes the total number of tests and N_{pool} denotes the total number of pools, the above equation simplifies to:

$$\mathbb{E}(T) = \frac{1}{n} + P\,(\text{positive})\,. \tag{3.2}$$

Similarly, the optimal pool size (n^* rounded to the nearest integer) is given as the pool size which minimizes $\mathbb{E}(T)$, hence the derivative of $\mathbb{E}(T)$ against n at n^* is zero:

$$\left.\frac{\partial}{\partial n}\mathbb{E}(T)\right|_{n=n^*} = 0. \tag{3.3}$$

Noticeably, Eq.2.1 is identical to Eq.3.2 after simplification, while their subsequent methods to solve this objective function are slightly different.

4 Algorithm based on H.Y. Kim et al. (2007) [2]

Suppose we have a population of size $P = \prod_{i=0}^{S-1} k_i,\ k_i \in \mathbb{Z}^+$, and the ith stage test has k_{i-1} pools of size n_i. For example, the first stage pooling will be k_0 pooled groups, each of size n_1. Therefore the pool sizes and numbers of pools have the following relationship:

$$n_i = \prod_{j=i}^{S-1} k_j. \tag{4.1}$$

We also define X_{si} as the random variable representing the test result of the ith pool at the sth stage, such that:

$$X_{si} = \begin{cases} 1 & i^{\text{th}} \text{ pool tests positive at stage } s \\ 0 & i^{\text{th}} \text{ pool tests negative at stage } s \end{cases} \tag{4.2}$$

Then, the expected number of tests given n_1 individuals, $\mathbb{E}(t)$, is given by:

$$\mathbb{E}(t) = 1 + \sum_{j=1}^{S-1} \mathbb{E}(X_{ji}) \prod_{i=1}^{j} k_i. \tag{4.3}$$

Based on [5], the expectation of X_{si} can be given as:

$$\begin{aligned}\mathbb{E}(X_{si}) = {} & (1-p)^{n_1}(1-S_p)^s \\ & + \sum_{j=1}^{s-1} \left[(1-p)^{n_{j+1}} - (1-p)^{n_j}\right] S_e^j (1-S_p)^{s-j} \\ & + \left[1-(1-p)^{n_s}\right] S_e^s,\end{aligned} \tag{4.4}$$

for $s = 1, 2, \cdots, S-1$. Therefore, for a two-staged test (i.e. $S = 2$), the expected number of tests per individual is given by:

$$\mathbb{E}(T) = \mathbb{E}\left(\frac{t}{n}\right) = \frac{1}{n} + (1-S_p)(1-p)^n + S_e\left[1-(1-p)^n\right]. \tag{4.5}$$

Hence the optimal pool size is given by:

$$n^* = \arg\min_{n>1} \mathbb{E}(T). \tag{4.6}$$

5 Algorithm based on R. Hanel et al. (2020) [4]

R. Hanel et al. provides a method to control false negatives of the pooled test by replicating the pool r times. If the majority of the replicates register as positive, the pooled sample is declared positive, and vise versa. Therefore, the probability of a pooled test falsely declaring positive $P_+^{(r)}$ and negative $P_-^{(r)}$ are given by:

$$P_+^{(r)} = \sum_{i \geq r/2} \binom{r}{i} (1 - S_p)^i (S_p)^{r-i}, \tag{5.1}$$

$$P_-^{(r)} = \sum_{i > r/2} \binom{r}{i} (1 - S_e)^i (S_e)^{r-i}, \tag{5.2}$$

then the probability P_+^*, that a test with r replicates registers as positive is given by:

$$P_+^*(r) = P\,(\text{positive}) \left(1 - P_-^{(r)}\right) + (1 - P\,(\text{positive}))\, P_+^{(r)}, \tag{5.3}$$

and $P_+^*(r = 1)$ is the probability for a single test without replicates to be registered as positive. Hence, the expected number of tests per person $\mathbb{E}(T)$ is given by:

$$\mathbb{E}(T) = \frac{1}{n}[r(1 - P_+^*(r)) + (r + n)\, P_+^*(r)] = P_+^*(r) + \frac{r}{n}, \tag{5.4}$$

which, when $r = 1$, can be equivalently expressed as:

$$\begin{aligned} \mathbb{E}(T) = {} & \frac{1}{n} + (1 - S_p)(1 - p)^n + S_e [1 - (1 - p)^n] \\ & + (S_e + S_p - 1) \sum_{k=1}^{n} \binom{n}{k} p^k (1 - p)^{n-k} (1 - S_e)^k S_p^{n-k}. \end{aligned} \tag{5.5}$$

Note that the first row of Eq.5.5 is identical to Eq.4.5. The optimal pool size is given by solving the derivative of the number of individuals per test N_{ppt} equals zero:

$$\frac{\partial}{\partial n} N_{ppt} = -N_{ppt}^2 \left(-\ln(1 - p)^{n+1} \left(1 - P_+^{(r)} - P_-^{(r)}\right) - \frac{r}{n^2}\right) = 0, \tag{5.6}$$

which leads to:

$$n^2 e^{\ln(1-p)n} = \frac{r}{\left(1 - P_+^{(r)} - P_-^{(r)}\right) \ln\left(\frac{1}{1-p}\right)}, \tag{5.7}$$

and further,

$$\frac{n}{2}\ln\left(1-p\right)e^{\frac{n}{2}\ln(1-p)}=\frac{1}{2}\ln\left(1-p\right)\sqrt{\frac{r}{\left(1-P_{+}^{(r)}-P_{-}^{(r)}\right)\ln\left(\frac{1}{1-p}\right)}}. \tag{5.8}$$

Hence, we obtain the optimal pool size n^* as:

$$n^{*}=\frac{W_{0}\left(\frac{1}{2}\ln\left(1-p\right)\left(\frac{r}{\left(1-P_{+}^{(r)}-P_{-}^{(r)}\right)\ln\left(\frac{1}{1-p}\right)}\right)^{\frac{1}{2}}\right)}{\frac{1}{2}\ln\left(1-p\right)}, \tag{5.9}$$

where W_0 is the principal branch of the Lambert-W function.

References

[1] Diego Aragón-Caqueo, Javier Fernández-Salinas, and David Laroze. Optimization of group size in pool testing strategy for sars-cov-2: A simple mathematical model. *Journal of Medical Virology*, 92(10):1988–1994, 2020.

[2] Hae-Young Kim, Michael G Hudgens, Jonathan M Dreyfuss, Daniel J Westreich, and Christopher D Pilcher. Comparison of group testing algorithms for case identification in the presence of test error. *Biometrics*, 63(4):1152–1163, 2007.

[3] Francesca Regen, Neriman Eren, Isabella Heuser, and Julian Hellmann-Regen. A simple approach to optimum pool size for pooled sars-cov-2 testing. *International Journal of Infectious Diseases*, 100:324–326, 2020.

[4] Rudolf Hanel and Stefan Thurner. Boosting test-efficiency by pooled testing for sars-cov-2—formula for optimal pool size. *PLoS One*, 15(11):e0240652, 2020.

[5] Norman L Johnson, Samuel Kotz, and Xi-Zhi Wu. *Inspection errors for attributes in quality control*. CRC Press, 2020.
