# Supplementary material for: Optimal pooling strategies for respiratory virus testing: A comparative cost-effectiveness analysis
Source: PLOS Glob Public Health. 2026 Jul 16;6(7):e0006646. doi: 10.1371/journal.pgph.0006646 (PMC13375041; doi:10.1371/journal.pgph.0006646)
Supplement: S2 File — (PDF) [file pgph.0006646.s002.pdf]

# Supplementary File 2

According to Baha Abdalhamid et al. [1], the costs of polymerase chain reaction (PCR) testing can be categorized into reagents and consumables, and labor costs. As our study is aimed at pooling during the collection process, we do not incorporate the cost to set up for original pools. Therefore, the total costs for a single stage of testing can be given by:

$$\begin{aligned} C_{total} =& (C_{RNA} + C_{PCR}) \times N_{test} + (t_{RNA} + t_{PCR}) \times C_{labor} \times N_{test} \\ &+ \ t_{report} \times C_{labor} \times N_{sample} + t_{collection} \times C_{labor} \times N_{sample}, \end{aligned}$$

where $N_{test}$ denotes the number of PCR tests conducted and $N_{sample}$ denotes the number of samples (or specimens) involved. We determine the value for $\alpha_2$ through dividing the mean cost per individual of second stage testing by that of the first stage:

$$\alpha_2 = \frac{C_{total,\ stage\ 2}/N_{sample,\ stage\ 2}}{C_{total,\ stage\ 1}/N_{sample,\ stage\ 1}}.$$

Table 1 below shows the universal input values for the parameters, which we use along with the values of $N_{test}$ and $N_{sample}$ to compute the values of $\alpha_2$ within each set of our simulation trial.

Table 1: Input values for parameters and their sources

| **Parameter** | **Description** | **Value** | **Source** |
|---|---|---|---|
| $C_{RNA}$ | unit cost for RNA extraction kit | \$9.18 | [1] |
| $C_{PCR}$ | average cost for RT-PCR consumables | \$5.48 | [1] |
| $t_{RNA}$ | average time required to extract one sample | 0.0357 h | [1] |
| $t_{PCR}$ | average time required to run one PCR test | 0.0178 h | [1] |
| $C_{labor}$ | average PCR technologist salary plus benefits | \$36.50/h | [1] |
| $t_{report}$ | average time to report per specimen | 1.5 min | [1] |
| $t_{collection,\ stage\ 1}$ | average collection time per specimen during first-stage testing | 15 s | [2] |
| $t_{collection,\ stage\ 2}$ | average collection time per specimen during second-stage testing | 0.02 h | [3] |

# References

[1] Baha Abdalhamid, Christopher R Bilder, Jodi L Garrett, and Peter C Iwen. Cost effectiveness of sample pooling to test for sars-cov-2. *Journal of infection in developing countries*, 14(10):1136, 2020.

[2] Centers for Disease Control and Prevention. Interim guidelines for collecting and handling of clinical specimens for COVID-19 testing. Web Page, October 29 2024. Accessed: February 22, 2026.

[3] Zha Qinghua, Wang Xiaoning, Zhang Yin, Ni Xueping, Chen Yan, and Zhu Weiyi. Exploration and practice of nucleic acid sampling outside hospital during the epidemic period of covid-19 pandemic. *Journal of Diagnostics Concepts & Practice*, 21(02):229, 2022.
