# Supplementary material for: Optimal pooling strategies for respiratory virus testing: A comparative cost-effectiveness analysis
Source: PLOS Glob Public Health. 2026 Jul 16;6(7):e0006646. doi: 10.1371/journal.pgph.0006646 (PMC13375041; doi:10.1371/journal.pgph.0006646)
Supplement: S3 File — (PDF) [file pgph.0006646.s003.pdf]

Supplementary File 3

As shown in the Results section of the main article, the algorithm provided by H.Y. Kim et al. (2007) [1] caused error when the disease prevalence p was close to 0.3, and both Polymerase Chain Reaction (PCR) testing sensitivity S_e and specificity S_p were low. This supplementary file is aimed to explain why such abnormal results were generated. By recalling the Eq.4.5 in Supplementary File 1, the objective function for H.Y. Kim et al.'s algorithm is given by:

$$\mathbb{E}\left(T\right) = \frac{1}{n} + \left(1 - S_p\right)\left(1 - p\right)^n + S_e\left[1 - \left(1 - p\right)^n\right], \tag{1}$$

with the optimal pool size n^* found by:

$$n^* = \arg\min_{n>1} \mathbb{E}(T). \tag{2}$$

To explain why n^* became larger with conditions of higher p, lower S_e and lower S_p, we derive from the behavior of the objective function. For clearer and simpler reference below, we define the last two terms in the objective function as the false positive (FP) term and the true positive (TP) term:

$$\mathbb{E}\left(T\right) = \frac{1}{n} + \underbrace{\left(1 - S_p\right) f(n)}_{\text{FP term}} + \underbrace{S_e\left[1 - f(n)\right]}_{\text{TP term}}, \tag{3}$$

where $f(n) = \left(1 - p\right)^n$.

1 The $\frac{1}{n}$ term

No matter how p, S_e and S_p changes, the first term $\frac{1}{n}$ decreases for larger pool size n. Therefore it always favors larger n^*.

2 The FP term

Since $p \in (0, 1)$, $f(n)$ decreases as n becomes larger. As a result, the FP term decreases with the rise of n, favoring larger n^*.

At lower S_p, the coefficient $(1 - S_p)$ becomes larger, making this FP term contributing more to the total $\mathbb{E}\left(T\right)$ and hence further pushing n^* larger.

3 The TP term

The value of $f(n)$ drops asymptotically as n increases, and this behavior becomes more obvious at higher p (see Figure 1). Therefore, the difference in $f(n)$ between small and large n values is less significant at higher p value, leading to a smaller difference in this TP term.

Hence, even though the TP term naturally increases as n becomes larger and hence should favor smaller n^* intuitively, larger n^* in fact raises the TP term by a rather small degree, which makes large n still possible to minimize $\mathbb{E}(T)$. Therefore, larger n^* may occur.

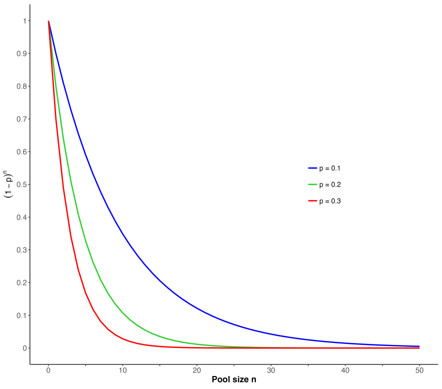

Figure 1: Plot of $f(n)$ against n

At lower S_e, this TP term contributes even less to $\mathbb{E}(T)$, which further diminishes the increase of this term with larger n^*.

4 An additional comment on the FP and TP terms

As Supplementary Figure 3 in the main article shown (Figure 2 below), n^* is much more sensitive to decrease in S_e than in S_p. This is largely caused by the behavior of $f(n)$ that $f(n) < 1 - f(n)$, leading to much smaller coefficient of S_p than S_e.

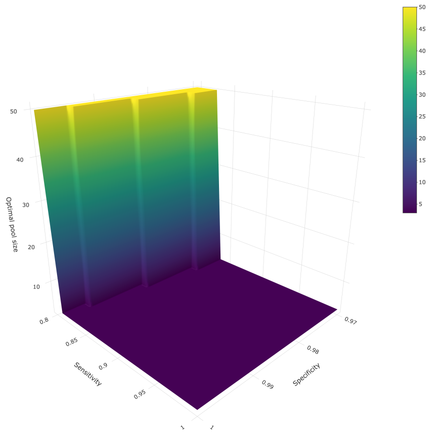

Figure 2: Surface plot of n against S_e and S_p at $p = 0.27$ (identical to Supplementary Figure 2 in the main article)

References

[1] Hae-Young Kim, Michael G Hudgens, Jonathan M Dreyfuss, Daniel J Westreich, and Christopher D Pilcher. Comparison of group testing algorithms for case identification in the presence of test error. *Biometrics*, 63(4):1152–1163, 2007.
