# Supplementary figures and images for: Optimal pooling strategies for respiratory virus testing: A comparative cost-effectiveness analysis
Source: PLOS Glob Public Health. 2026 Jul 16;6(7):e0006646. doi: 10.1371/journal.pgph.0006646 (PMC13375041; doi:10.1371/journal.pgph.0006646)

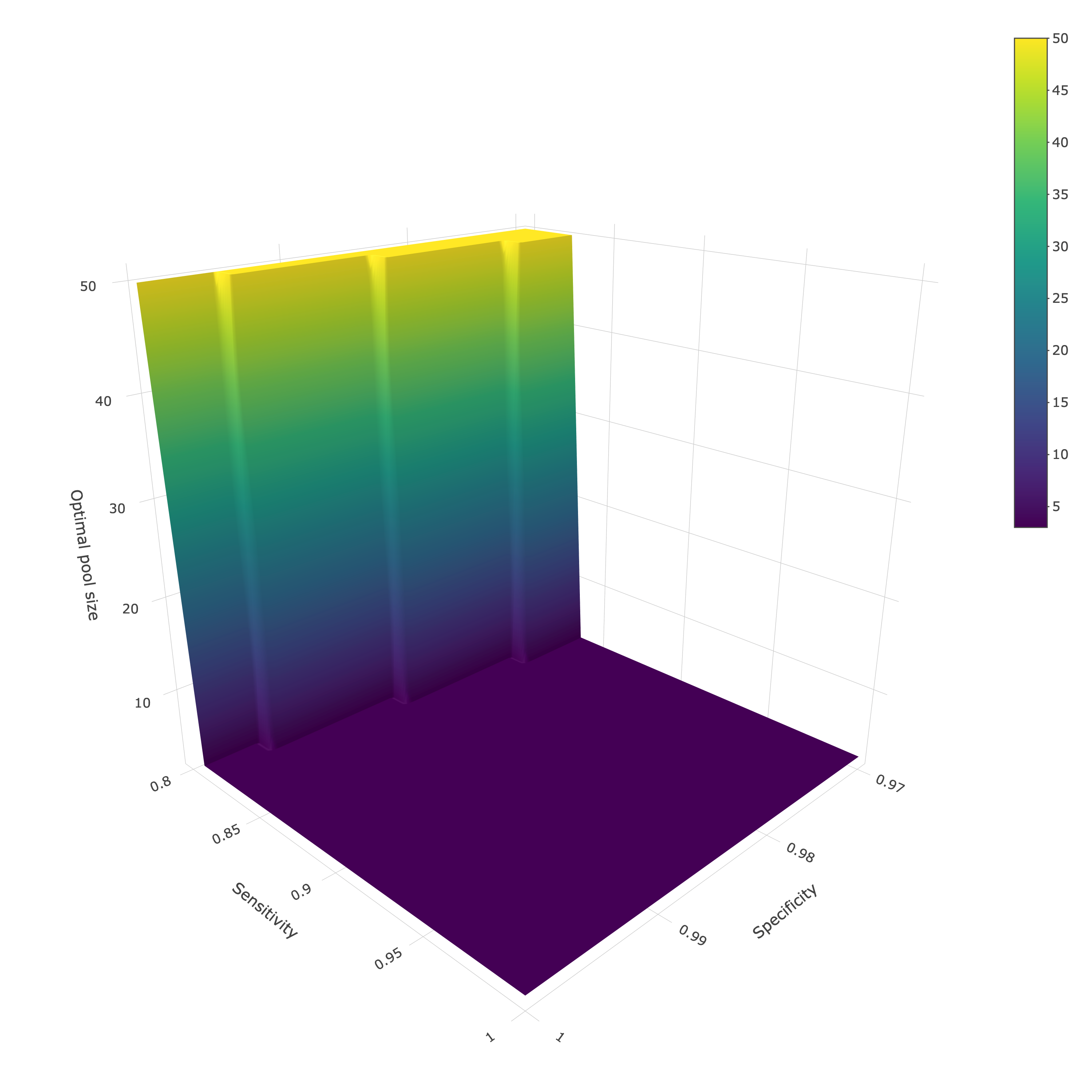

Supplement: S2 Fig — (TIF) [file pgph.0006646.s005.tif]

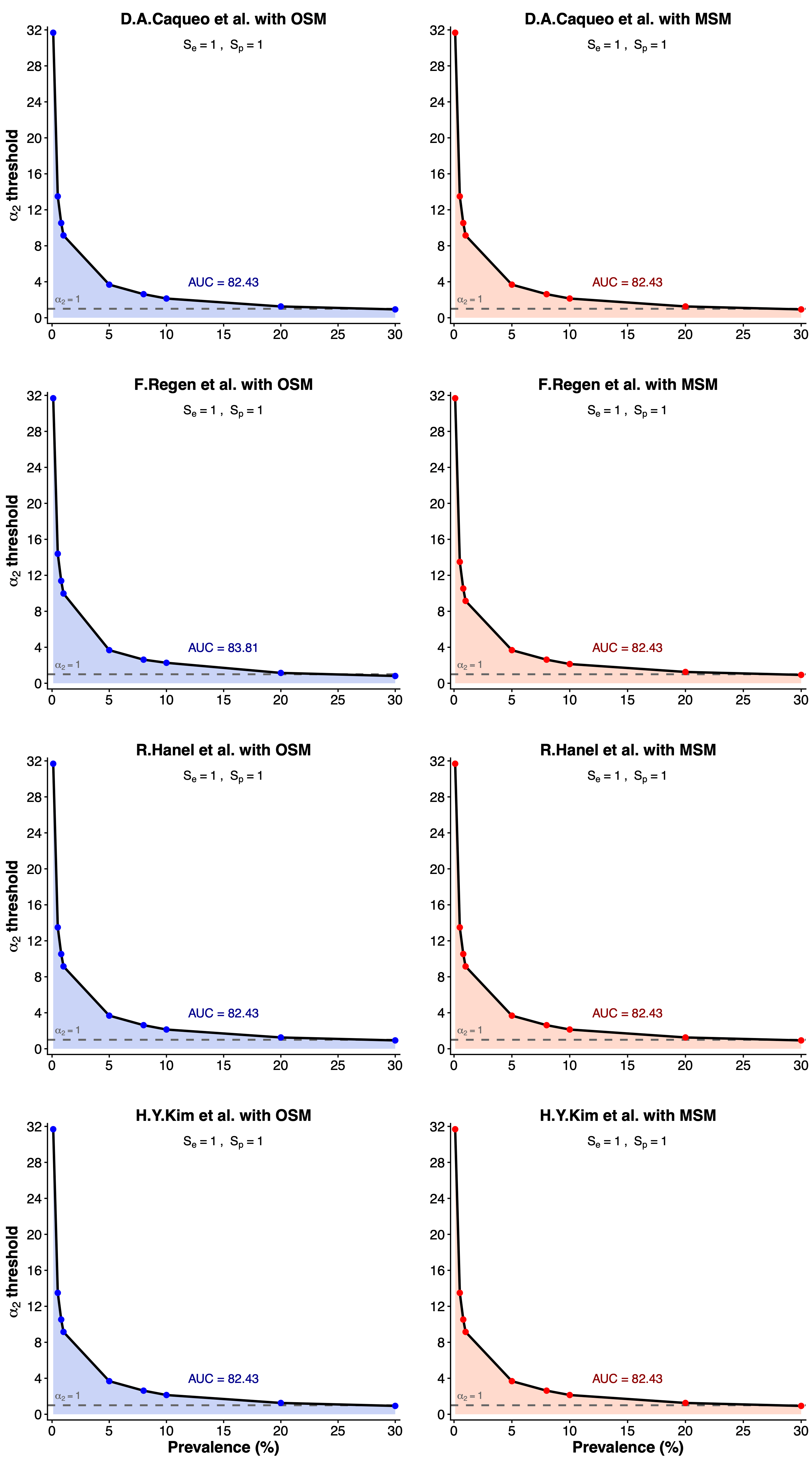

Supplement: S4 Fig — (TIF) [file pgph.0006646.s007.tif]

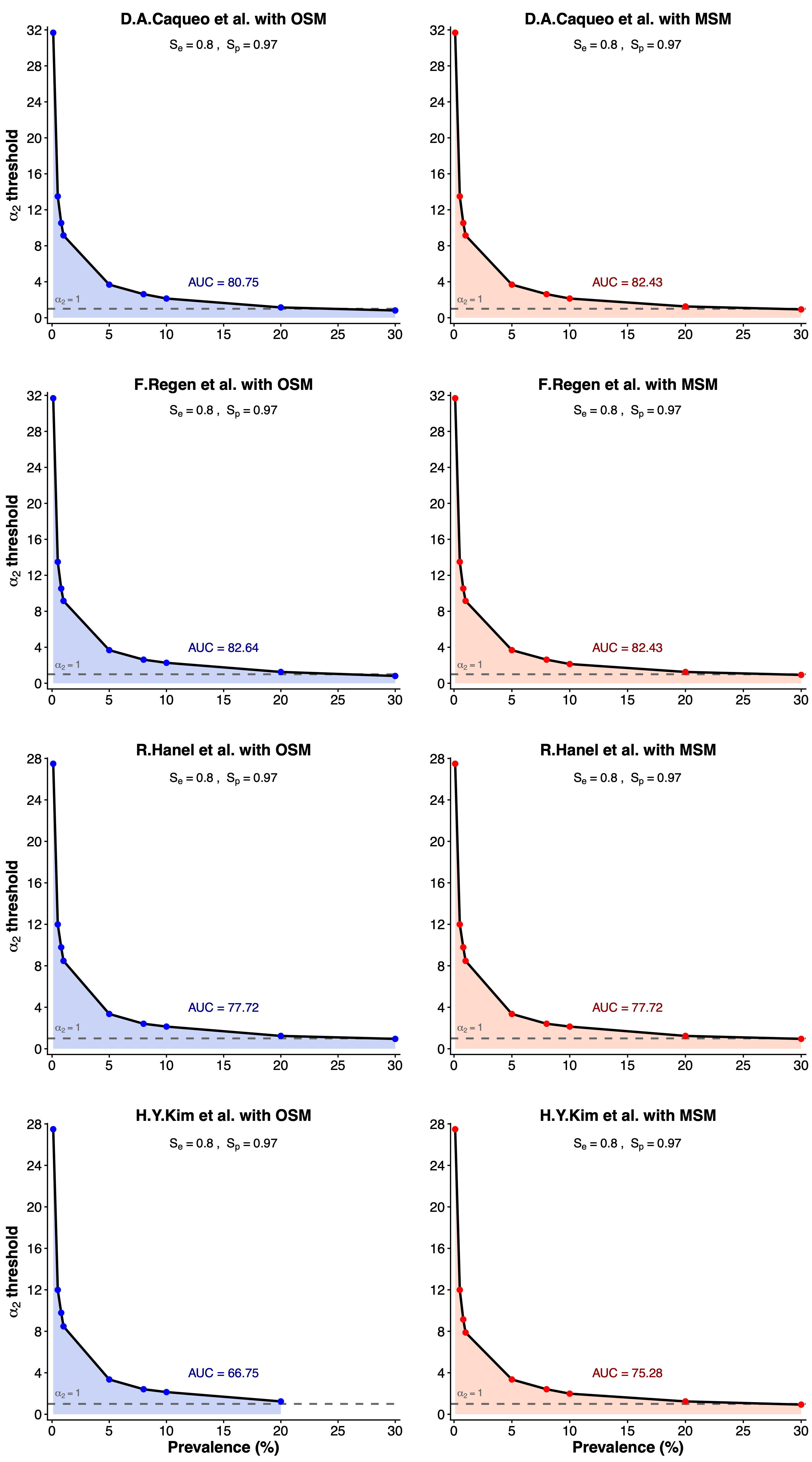

Supplement: S5 Fig — (TIF) [file pgph.0006646.s008.tif]
