# Supplementary material for: Optimal pooling strategies for respiratory virus testing: A comparative cost-effectiveness analysis
Source: PLOS Glob Public Health. 2026 Jul 16;6(7):e0006646. doi: 10.1371/journal.pgph.0006646 (PMC13375041; doi:10.1371/journal.pgph.0006646)
Supplement: S1 Table — (DOCX) [file pgph.0006646.s010.docx]

**Supplementary Table 1. Bisection procedure to determine intercept of a logit model for outcomes with various expected prevalences, all with initial intervals of (-10, 10)**

| Max iteration No. | Expected prevalence | $\beta_{0}$^midpoint^ | Empirical prevalence |
| --- | --- | --- | --- |
| 36 | 0.001 | -6.899297 | 0.00100 |
| 18 | 0.005 | -5.275192 | 0.00497 |
| 12 | 0.008 | -4.799805 | 0.00807 |
| 9 | 0.010 | -4.570312 | 0.00996 |
| 10 | 0.050 | -2.949219 | 0.04984 |
| 10 | 0.080 | -2.441406 | 0.08010 |
| 6 | 0.100 | -2.187500 | 0.10099 |
| 10 | 0.200 | -1.386719 | 0.20019 |
| 10 | 0.300 | -0.839844 | 0.30161 |
