# Supplementary material for: Optimal pooling strategies for respiratory virus testing: A comparative cost-effectiveness analysis
Source: PLOS Glob Public Health. 2026 Jul 16;6(7):e0006646. doi: 10.1371/journal.pgph.0006646 (PMC13375041; doi:10.1371/journal.pgph.0006646)
Supplement: S2 Table — (DOCX) [file pgph.0006646.s011.docx]

**Supplementary Table 2. OPSs obtained from four algorithms using OSM or MSM at various prevalences when sensitivity=1 and specificity=1**

| Prevalences (%) | D.A. Caqueo et al. | | F. Regen et al. | | H.Y. Kim et al. | | R. Hanel et al. | |
| --- | --- | --- | --- | --- | --- | --- | --- | --- |
|  | OSM | MSM | OSM | MSM | OSM | MSM | OSM | MSM |
| 0.1 | 32 | 32 | 32 | 32 | 32 | 32 | 32 | 32 |
| 0.2 | 23 | 23 | 22 | 23 | 23 | 23 | 23 | 23 |
| 0.5 | 15 | 15 | 14 | 15 | 15 | 15 | 15 | 15 |
| 0.8 | 12 | 12 | 11 | 12 | 12 | 12 | 12 | 12 |
| 1.0 | 11 | 11 | 10 | 11 | 11 | 11 | 11 | 11 |
| 2.0 | 8 | 8 | 7 | 8 | 8 | 8 | 8 | 8 |
| 5.0 | 5 | 5 | 5 | 5 | 5 | 5 | 5 | 5 |
| 8.0 | 4 | 4 | 4 | 4 | 4 | 4 | 4 | 4 |
| 10.0 | 4 | 4 | 3 | 4 | 4 | 4 | 4 | 4 |
| 20.0 | 3 | 3 | 2 | 3 | 3 | 3 | 3 | 3 |
| 30.0 | 3 | 3 | 2 | 3 | 3 | 3 | 3 | 3 |
