# Supplementary material for: Optimal pooling strategies for respiratory virus testing: A comparative cost-effectiveness analysis
Source: PLOS Glob Public Health. 2026 Jul 16;6(7):e0006646. doi: 10.1371/journal.pgph.0006646 (PMC13375041; doi:10.1371/journal.pgph.0006646)
Supplement: S3 Table — (DOCX) [file pgph.0006646.s012.docx]

**Supplementary Table 3. Parameters, input values and sources for cost-effectiveness analysis**

| Parameters | Inputs | Sources |
| --- | --- | --- |
| Disease prevalence | Multiple values in range 0.1% - 30.0% | Ref ^22^ |
| PCR testing sensitivity | Multiple values in range 80% - 100% | Ref ^7^ |
| PCR testing specificity | Multiple values in range 97% - 100% | Ref ^7^ |
| Relative cost coefficient of first-stage testing $\alpha_{1}$ | 1.0 | Assumed |
| Relative cost coefficient of second-stage testing $\alpha_{2}$ | See Supplementary File 2 | Ref ^30^ |
